# Persons With Schizophrenia Misread Hemingway: A New Approach to Study Theory of Mind in Schizophrenia

**DOI:** 10.3389/fpsyt.2020.00396

**Published:** 2020-05-07

**Authors:** Judit Fekete, Zsuzsanna Pótó, Eszter Varga, Tímea Csulak, Orsolya Zsélyi, Tamás Tényi, Róbert Herold

**Affiliations:** ^1^Doctoral School of Clinical Neurosciences, Medical School, University of Pécs, Pécs, Hungary; ^2^Department of Behavioural Sciences, Medical School, University of Pécs, Pécs, Hungary; ^3^Department of Pediatrics, Medical School, University of Pécs, Pécs, Hungary; ^4^Department of Psychiatry and Psychotherapy, Medical School, University of Pécs, Pécs, Hungary

**Keywords:** schizophrenia, Theory of Mind, ToM, reading, mental state reasoning, literature, Short Story Task

## Abstract

**Introduction:**

Theory of Mind (ToM) is a key component of social cognition. Recently the Short Story Task (SST) was developed as a new measurement of ToM. SST uses a short story of Ernest Hemingway to assess ToM skills. SST proved to be a suitable tool, and sensitive to individual differences among healthy subjects. Our aim was to test SST to evaluate the ToM skills of persons with schizophrenia.

**Materials and Methods:**

SST was used to assess ToM skills. After reading the short story “The End of Something” a structured interview was done with 14 questions. Spontaneous mental state reasoning, explicit mental state inference and comprehension of nonmental aspects of the story were evaluated. 47 persons with schizophrenia in remission and 48 healthy controls were assessed and compared.

**Results:**

Persons with schizophrenia performed significantly more poorly in the explicit mental state inference questions. Ceiling effect was not detectable in explicit ToM scores. Patients made less spontaneous mental state references as well, although the occurrence of spontaneous mental state terms was infrequent in both groups. Patients were also less accurate in answering comprehension questions, but the difference was not significant after Bonferroni correction.

**Discussion:**

Our results lined up with the original findings and we found SST to be a sensitive tool to explore the individual differences in ToM performance, not only among healthy subjects, but also among persons with schizophrenia especially in explicit mental state inferences without observing the ceiling effect. We found, however, SST to be less sensitive to measure spontaneous mental state reasoning and also the lack of the use of another ToM test to assess convergent validity of SST for indicating ToM deficits in schizophrenia stands as a limitation of current study.

## Introduction

Literature, especially literary fiction, is an excellent way of practicing Theory of Mind (ToM), since it is characterized by using narrative content that prompts the reader to guess rather complex social situations, where the motivation of the characters and/or the causality of the events are not described directly or explicitly. The narrative comprehension of these scenarios usually requires the use of ToM skills, because the reader is required to make mental state inferences to understand the events and the characters' intentions. An interesting approach has been published recently that suggested literary fiction as a potential tool to assess ToM performance (1) and they used a short story to assess ToM skills (Short Story Task, SST) (1).

ToM is the ability to attribute mental states (such as beliefs, knowledge, intentions, emotions) to the self and others, and hence it allows to explain and predict behavior (2, 3) while being a key component of social cognition. Theory of mind has two major components: its implicit and its explicit form. Implicit ToM is automatic, fast, decoded without awareness, non-verbal, and so it is an intuitive mental state attribution (4). The implicit ToM is present in the early life, possibly from birth (5, 6). Explicit ToM, in contrast, is relatively slow, relying on verbal processing, more controlled and conscious, deliberative, and inferential (4, 7). Compared to implicit ToM, explicit ToM develops later, and closely tied to language acquisition and executive function development (5). Explicit ToM is usually measured by tasks relying heavily on verbal abilities and they usually contain explicit instructions for attributing mental states (8). The term, spontaneous ToM is also used in social cognitive research. Spontaneous ToM is the processing of social information without explicit instruction (9). The concept of spontaneous ToM overlaps with the implicit ToM, however according to Senju (9), spontaneous ToM does not require the lack of conscious awareness. It is also not an obligatory processing unlike automatic processing, so it can be interrupted by competing cognitive tasks (9). It can be conceived as a lower level and early phase ToM processing with an emphasis on mental state decoding (4). Usually the spontaneous ToM activity is tested with animated geometric forms stimuli, and measured by multiple choice questions (8, 10, 11), or with question not directly asking the subject to reflect upon mental states (7). These types of ToM tasks are also used as indirect measures of verbal mental state attribution indexed by the spontaneous use of mental-state language (7) (e.g. feels, thinks, wonders, furious, anxious, etc.) in tasks when the participants are not cued (unlike in explicit ToM tasks), and responses are spontaneous (1, 7).

The concept behind the idea to use literary fiction as an assessment tool was to make a test sensitive to spontaneous and explicit ToM as well as to small individual differences so that the ceiling effect could not be detected. Ceiling effect, when participants perform near perfectly, is quite frequent in ToM research. They also considered it important to include a range of complexity tasks in order to use a social situation similar to reality and to be easily and quickly recruited and evaluated. Literary fiction is very similar to everyday situations: it is about a dynamically developing, complicated, lifelike social situation, where the understanding of the mental aspects is essential for the comprehension of the entire story. In order for the reader to interpret the story, one must draw conclusions about the thoughts, emotions and intentions of the characters just as in everyday situations (1).

A substantial body of evidence suggests, that persons with schizophrenia are impaired in their abilities to attribute mental states to others (12–14). Research results confirmed that ToM impairment does not only exist during acute episodes as a state variable, but it is also constantly present between the episodes as trait-marker (12). ToM deficits in schizophrenia could be conceived as a phenotypic impairment (13, 15), it precedes the onset of the disorder, and it is not only present in the acute psychotic states, but also during remission (13). High-risk individuals show lower ToM performance with blunted trajectory from age 17 onward (16). It can be also detected in nonaffected relatives with genetic risk, they usually have intermediate performance between persons with schizophrenia and healthy controls (13, 17). Genetic associations with ToM deficits were also revealed during the last decade (13). According to literary data, the resulting ToM deficit is independent of age and gender (14) and—although not specifically addressed so far—an association of ToM impairment with medication seems at least unlikely (18). Cross cultural studies also revealed that ToM impairment in schizophrenia is present across cultures (11, 19). It is also assumed that not only the understanding of others is impaired, but patients also have a disturbed capacity to relate their own intentions to executing behavior, and to monitor others' intentions (20).

Most available research studying schizophrenia investigated the explicit ToM skills, which were found extensively deficient. Relatively few studies focused on implicit and spontaneous ToM. Roux et al. found (21) preserved implicit mental-state attribution measured by eye-tracking, whereas explicit performance was impaired, however, they also detected slowdown of social context processing during intention attribution with a similar paradigm (22). Other investigations—which used animated geometric forms stimuli—found deficient spontaneous ToM skills characterized with incorrect social inferences in schizophrenia (8, 10, 11, 23). Other studies with animated geometric forms reported fewer patient generated mental-state terms (7, 24, 25). The reduced unprompted mention of mental states reflected a relative insensitivity to salience of mental state information.

There is a strong link between social cognition, functional outcomes and quality of life (26, 27). In schizophrenia, ToM deficits are difficult to overcome and improve, while adequate social cognitive abilities are indispensable for proper social functioning. In light of the importance of social intelligence including mental state attribution skills in human evolution, it seems straightforward to assign mental state attribution a specific role in social functioning (28).

In the past decades, numerous tests have been developed to measure ToM. Overall it can be stated, that there are so many tests and methods, which successfully examine and measure ToM, that it would go beyond the scope of this article to give a fully detailed listing on them. The different methods were reviewed excellently in several publications [for detailed review see (1, 29, 30)]. These measures and other methods of analysis usually clearly distinguish persons with autism, bipolar disorder, schizophrenia, etc. from the healthy population based on their marked ToM deficits. Most of these tasks, however, were primarily developed for children and may not be challenging enough to assess adults. These tests could often only detect serious impairments, and the tiny individual differences—even among healthy individuals—would remain hidden. The additional disadvantage of these methods is that members of the healthy control groups often perform 100% or nearly 100% so ceiling effect can be detected. Ceiling effect is a common phenomenon in ToM research in schizophrenia as well, when healthy controls are involved in a study, as they usually perform above 90% in several studies (1, 30). Even early detection of the slightest deficit could, however, significantly advance preventive and diagnostic activities. Unaffected first-degree relatives or individuals with high-risk state for psychosis usually exhibit milder forms of ToM impairments (31), but even persons with schizophrenia exhibit varying degrees of ToM deficits (28).

Additionally, the greater part of ToM studies are limited to studying explicit ToM so the participant is instructed to make ToM references. In these tests, spontaneous ToM, where the participant is not specifically instructed to make references for mental states, is usually ignored (1), however recent studies addressing spontaneous ToM have been emerging (7, 8, 10, 11, 23–25).

The main purpose of this study was to test the applicability of the new SST (1) to measure the ToM skills of persons with schizophrenia. It was hypothesized that significant differences would be detected in the explicit ToM scores between the persons with schizophrenia and the control group participants. We also presumed that there would be significantly more spontaneous ToM references among healthy subjects than among patients. We also hypothesized, that there would be no significant differences in the participants' comprehension skills in terms of the short story. Finally, based on the previous results, we did not expect to observe a ceiling effect in mental state reasoning in both groups.

## Material and Methods

### Participants

The persons with schizophrenia (schizophrenia group, SG) were recruited from outpatient psychiatric services and from the outpatient units of inpatient psychiatric cares from three cities (Pécs, Mohács and Szigetvár) in Hungary. All the patients were treated with the diagnosis of schizophrenia fulfilling the diagnostic criteria of DSM-5. Two experienced, senior psychiatrists reviewed the psychiatric history of the patients to confirm the diagnosis. Diagnosis was also confirmed by Module B and C of SCID-5 (Module B: Psychotic Symptoms, Module C: Differential Diagnosis of Psychotic Disorders) (32). Patients were on maintenance antipsychotic treatment. Patients received first generation antipsychotics (8 persons), second generation antipsychotics (28 persons), or they were on combination treatment with two antipsychotics (11 persons). The chlorpromazine equivalent dose was 371.21 mg (SD: 201.62). Inclusion criteria were: age older than 18; being native Hungarian speaker; no evidence of substance abuse (excluding caffeine and tobacco); no neurological disorder or mental retardation or cognitive deficits unrelated to schizophrenia. All the subjects living with schizophrenia were treated as outpatients, there were no changes in the medication of the participants during the study and in the antecedent last 6 months. We intended to assess a clinically stable patient population fulfilling the criteria of remission to minimize the confounding effect of symptoms. According to the remission criteria of schizophrenia (33), remission was confirmed with the eight items (P1, P2, P3, N1, N4, N6, G5, G9) of Positive and Negative Syndrome Scale (PANSS), which were mild or less (≤3) for at least 6 months before entering the assessment. Sixty two patients with the diagnosis of schizophrenia in a clinically stable state according to the judgement of their treating psychiatrist were recruited. Fifteen patients were ruled out, as they were not in remission according to the remission criteria of schizophrenia. The final sample comprised of 47 subjects (23 males and 24 females).

The control group (CG) consisted of 48 Hungarian-speaking healthy individuals (19 males and 29 females), enrolled from the general community through online recruitment. Inclusion criteria for the CG were the following: age older than 18; being a native Hungarian speaker; no evidence of substance abuse (excluding caffeine and tobacco); no neurological disorder, no earlier treatment due to psychiatric disorder. CG was also screened with SCID-5 to exclude the presence of a psychiatric disorder. Age, sex, ethnic origin and educational status were matched to the characteristics of the patients' group to minimize interindividual variability.

The psychiatric history review, the assessment of remission, and SCID-5 were carried out by two senior psychiatrists (R. Herold, T. Tényi) trained in SCID-5 and PANSS assessment. The interrater reliability of them was tested in our earlier study for SCID and PANSS, and the kappa coefficient was >0.75 (34).

After a detailed description of the study was presented to the subjects, written informed consents were obtained. Patients were aware of the study's aims and hypotheses. The investigation was performed according to institutional guidelines. Ethical perspectives were established in accordance with the latest version of the Declaration of Helsinki. The study design was approved by Committee on Medical Ethics, University of Pécs (ethical permit number: 6539).

### Experimental Task

For the present investigation, SST was used, previously developed by Dodell-Feder et al. (1) for ToM investigation in healthy participants. The SST consists of a short story and a Short Story Task Administration and Scoring Materials. This supplementary document involves instructions for the participants, the questions, and scoring instructions. The original English version of the supplementary document can be downloaded from the publishing site (https://journals.plos.org/plosone/article?id=10.1371/journal.pone.0081279).

The test was adapted to Hungarian. We translated the instructions, the questions and the evaluation criteria, then a bilingual native speaker was asked to translate it back to English considering the intercultural differences. ToM skills were analyzed through a structured interview after reading the short story. The instructions were modified on the basis of the ex-post recommendations of the US task force. The original study demonstrated that SST is sensitive to variations in ToM performance, it can be rated accurately by different raters, and SST showed convergent validity with other ToM measures (1).

Participants read a short story, The End of Something by Ernest Hemingway, which presented an interaction between a romantic couple, Nick and Marjorie. This particular short story has been chosen for this purpose because the text is easy to understand (1). We used a published Hungarian translation of the short story (35). Throughout the story the couple followed the stages of a breakup however the mental lives of the characters were not explicitly described so the reader was forced to make mental state inferences by picking up clues from the various nonverbal and indirect communication between the characters.

Before reading the story, the participants were given verbal instructions, then afterwards were asked a series of open-ended questions. They were allowed to refer back to the story as needed to eliminate memory demands. In these instructions, readers were asked to highlight the characters' thoughts, feelings and intentions. The investigator gave no feedback regarding the participant's responses.

The task was presented verbally by one of the investigators in the form of an interview in one session for all participants individually. Each interview was recorded, and the recorded data were scored by two independent investigators (it was done by J. Fekete, and E. Varga). The interrater reliability was tested, and the kappa coefficient was >0.90 in the pilot study. According to the original study, scoring was completed by the first author, using the transcripts, then 25% of the transcripts were chosen at random and scored by a second independent rater (1).

The structured interview of SST involved 14 questions regarding three areas: five comprehension questions, eight explicit mental state reasoning questions, and one question to assess spontaneous mental state inference (1). Comprehension questions were designed to measure the understanding of the nonmental state content, while explicit mental state reasoning questions assessed the mental state inferences, and the understanding of non-verbal or indirect communication. To assess spontaneous mental state inference question, participants were asked only one open-ended question: to summarize the story in their own words. In this particular question responses were coded according to the presence or absence of mental state inference, without drawing special attention to what the question actually measures.

The answers for the questions were scored from 0 to 2 (except from the spontaneous mental state inference question, which is scored from 0 to 1: absence or presence of mental state inference). Zero (0) point was given when the answer was incorrect or when there was no answer. 1 point was given when the response demonstrated partial understanding, when the participant needed questions to clarify or when the participant gave very few examples. Two points were given when the responses demonstrated full understanding and the experimenter gave more than two examples.

### Statistical Analysis

The IBM Statistical Package for the Social Science [SPSS; SPSS Inc., Chicago, IL, USA (36)] Statistics version 24 for Windows was used for statistical analysis. In the statistical analysis, as we made multiple comparisons according to Bonferroni correction, p <0.01 was considered significant. We used independent samples t-test, ANCOVA and non-parametric Mann–Whitney U test to calculate the differences between the persons with schizophrenia (SG) and the control group (CG) for clinical and demographic data. We used Chi-square test to examine gender difference between the two groups. We performed linear regression to assess the effects of demographic data on explicit ToM.

## Results

Demographic data are summarized in **Table 1**. There was no significant difference in age (p = 0.942, not significant, n.s.), years of education (p = 0.243, n.s.), and gender (p = 0.759).

**Table 1:** Demographic data in the CG and the SG.

	Control Group (CG) (n = 48)		Schizophrenia Group (SG) (n = 47)		
	Mean	S. D.	Mean	S. D.	p-value
Age (year)	43.88	19.38	43.64	11.30	*P* = 0.942^a^
Education (years)	12.98	2.43	12.38	2.52	*P* = 0.243^a^
Duration of illness (years)			12.46	2.32	

a Independent Samples t-test was used to calculate the differences between the groups Statistically significant: p < 0.01, Bonferroni correction.

Persons with schizophrenia performed—statistically by average—less accurately than control subjects in the comprehension questions, but it was not statistically proven after the Bonferroni correction had been performed (p = 0.050) (**Table 2**).

**Table 2:** Differences in task performance between CG and SG.

Experimental tasks	Control Group (CG) (n = 48)		Schizophrenia Group (SG) (n = 47)		p-value
	Mean	S. D.	Mean	S. D.	
Comprehension questions	8.42	1.76	6.55	2.34	*0.050^a^*
Explicit mental state reasoning questions	9.08	3.75	4.98	3.96	*<0.001^a^*
Spontaneous mental state inference question	0.27	0.54	0.09	0.28	*<0.001^a^*

a Mann–Whitney U test was used to calculate the differences between the groups.

Statistically significant: p < 0.01, Bonferroni correction.

In the other two measured factors, namely in the explicit mental state reasoning questions (p <0.001), and in the spontaneous mental state inference question (p <0.001), the control group achieved significantly higher scores than the persons with schizophrenia (**Table 2**).

Following this, the explicit ToM scores were analyzed using linear regression in the two different groups. Both models exist according to **Table 3**, as the global F test's ANOVA values are under 0.01, and the explanatory powers are higher than 0.3, prompting the investigators that the models are satisfactory to draw conclusions. According to our findings, explicit ToM is not significantly influenced by age (pc = 0.036, ps = 0.076), education (pc = 0.388, ps = 0.981) and gender (pc = 0.595, ps = 0.343). Out of the participants so few responded to the spontaneous mental state inference questions in both groups (SG 4, CG 11) that it was not statistically relevant to analyze the influence of age, education, and gender on spontaneous mental state inferences.

**Table 3:** The effects of age, education, gender on explicit ToM skills in the two groups.

	Control group (CG) (n = 48)		Schizophrenic group (SG) (n = 47)	
***Model***	*ANOVA p-value*	*Adjusted R Square*	*ANOVA p-value*	*Adjusted R Square*
	0.000	0.378	0.001	0.332
*Parameters*	*Unstandardized B coefficients*	*p-value*	*Unstandardized B coefficients*	*p-value*
Age (year)	−0.050	0.036	0.087	0.076
Education (years)	0.162	0.388	−0.005	0.981
Gender (0 = female)	0.474	0.595	1.074	0.343

Linear Regression.

We analyzed further only the between group differences in explicit mental state reasoning for the same reason. To compare our two groups' ToM skills independently of comprehension in terms of explicit ToM, we performed ANCOVA to provide the statistical significance value of whether there are statistically significant differences in explicit ToM between the two groups (SG and CG) when adjusted for comprehension. We found that there is a statistically significant difference between adjusted means (p = 0.002), and persons with schizophrenia achieved significantly lower scores than those of the control group (**Table 4**).

**Table 4:** The number of people in each group and the differences in explicit ToM between the two groups (CG and SG) when adjusted for comprehension.

Explicit ToM	Diagnoses		p-value
	SG (n = 47)	CG (n = 48)	
Mean	4.98	9.08	0.002^a^

a ANCOVA test was used to calculate the differences between the groups.

Statistically significant: p < 0.01, Bonferroni correction.

Analyzing the ceiling effect in explicit ToM, scores in SG were relatively normally distributed with a slight positive skew (skew = 0.82, kurtosis = −0.11) indicating an asymmetry in the distribution where by the majority of scores were on the left side of the distribution (reflecting that the majority of individuals received scores of 7 out of 16 possible points or lower). Importantly, there was substantial variation in results across individuals with scores ranging from 0 to 15 (possible scores = 0–16), and no indication of a ceiling effect (2.1% of participants scoring 16/16 or 15/16) with a mean score 5.0 ± 1.2.

While examining the CG in the same field we also found that the data was close to the normal distribution with negative kurtosis (skew = −0.03, kurtosis = −1,10), which is flatter than the normal curve (so similar persons reached points 5–12). There was substantial variation in results across individuals with scores ranging from 2 to 15 (possible scores= 0–16), and there was no indication of a ceiling effect (8.3% of participants scoring 16/16 or 15/16). Mean score was 9.1 ± 1.1.

## Discussion

In the present study we report our results gathered from utilizing the Short Story Task, which was originally designed and tested in English with healthy participants (1). In the present research our aim was to test the applicability of SST in schizophrenia. As far as we know, this is the first study in which the ToM performance of persons with schizophrenia has been investigated while using this method.

According to our hypothesis, significant differences were detected in the explicit ToM scores between the persons with schizophrenia and the control group participants. Explicit mental state reasoning was found not to be influenced by age, education, and gender. The scores also indicated the lack of ceiling effect in both groups. In line with our expectations, there were also significantly more spontaneous ToM references among healthy subjects than among patients. Although our assumption has been substantiated, unfortunately the case numbers were quite low. Only a few participants (SG: 4/47, CG: 11/48) answered the spontaneous mental state inference question in both groups while using mental state terms. The patient group performed more poorly in the comprehension questions, although after Bonferroni correction—the difference between the groups did not reach the significance level.

Generally, our results confirm previous data that explicit ToM is markedly impaired in schizophrenia, however, we cannot dismiss the occurrence of a more general language processing disturbance (e.g. verbal comprehension, pragmatic deficit) in terms of weak ToM performance (7, 37). Although comprehension scores did not differ significantly after Bonferroni correction the performance of the patients was still poor compared to the healthy controls. These results may reflect a more general language processing deficit, however, they line up with the findings of the original study of Dodell-Feder et al. (1), namely that ToM scores were unrelated to understanding the nonmental aspects of the story. They concluded firstly, that their structured interview questions successfully isolated ToM skills from the general reading abilities, and secondly that comprehension scores did not link with the other measures of social cognition used for concurrent validation of the SST. For further investigation, we compared our two groups' explicit mental state reasoning skills when adjusted for comprehension. Persons with schizophrenia still achieved significantly lower scores in explicit mental state reasoning questions than the control group participants. On the other hand, so few participants responded while using mental state terms in the spontaneous mental state inferences questions in both groups, that it was not statistically relevant to compare their performances accordingly.

The low number of spontaneous mental state inferences in both groups was an unexpected result, and this finding may reveal a potential limitation of SST. It suggests that SST may be more suitable for evaluating the explicit mental states, as only one question seems disproportionately scant for assessing spontaneous mental states compared to the 8 explicit mental state reasoning questions. This may also suggest that supplementing SST with an easily applicable test for measuring spontaneous ToM [e.g. Social Attribution Task, multiple-choice version, SAT-MC, (8, 10)] would create a more complex and detailed picture of ToM deficits in schizophrenia. It is in line with literature suggesting that multi-modal assessment should be used to explore the ToM deficits in schizophrenia (19). Overall it could be concluded, that the new short story method is easy to implement, requires no special conditions, takes about 25–30 min on average to read the story and answer the questions, and it is easy to score reliably. The detailed instructions, questions, and the anchoring points in the clearly defined point system simplify the evaluation process. Although SST is easy to administer, the full time demand (reading the short story, the interview, and scoring) is longer than in other ToM measures (e.g. the administration time of above-mentioned SAT-MC is approximately 10 min), which in turn may limit the everyday use in certain clinical circumstances.

An important result of our study was the confirmation of the lack of ceiling effect since according to our findings neither the patients, nor the healthy participants achieved 100%, unlike in the other frequently used ToM tests, so the distribution of the scores confirms the lack of ceiling effect. It is important to note on the other hand that patient scores also showed very high variability, confirming the test's sensitivity to individual differences in terms of ToM skills.

This substantial variation in performance may assist to generate a more subtle assessment of ToM skills. ToM is a key aspect of social cognition, which has strong relations to community functioning (38), hence, ToM is a proximal skill to functioning, and taking these individual differences into consideration may even help in customizing the rehabilitation plan in the future. Additionally, further development of this new evaluation method may open perspectives for further preventive screenings of the ToM deficit in high-risk groups. Reliable data suggests that ToM skills deteriorate in the early course of the disorder, as clinically high-risk subjects exhibit a blunted developmental trajectory in ToM skills from age 17 onward (16).

Future perspective of SST can be linked to a potential further development of SST Administration and Scoring Materials, which might be the most prominent strength of SST. It could easily be adapted to different short stories, which means that potentially several different SST batteries could be generated, which in turn may enable the repeated use of SST to monitor the changes in ToM abilities. It should also be mentioned on the other hand that the relevance of literary fiction in ToM research expands beyond testing. According to recent results, fiction and narrative processing is an emerging and promising approach in improving social cognition (39). Reading literary fiction leads to better performance in ToM tests in healthy subjects compared to reading non-fiction, popular fiction, scientific fiction or nothing at all (40, 41). Most recently a meta-analysis found a small positive impact of fiction reading on social cognition (42). This impact could be linked to a characteristic feature of literary fiction, as it requires the reader to be engaged in a simulated social experience by being immersed in the mental and social life of the fictional characters (1, 43). Literary fiction can also bolster the learning of mental state vocabulary (44), which seems to be compromised in schizophrenia (7). Although there is no research data on the effect of literary fiction on ToM among persons with schizophrenia, there is a substantial tradition in using literature in psychosocial interventions (poetry therapy, bibliotherapy) as a facilitating tool to enhance social skills (45). Since reading fiction could promote ToM abilities, it could be conceived as a low-cost tool to be potentially integrated into psychosocial interventions or utilized in rehabilitation programs (41) in schizophrenia, leading us to a possible conclusion that SST could be a useful and natural equipment in measuring ToM skills and in their follow up examinations.

### Limitations

However, some limitations should be addressed. Such as the lack of full PANSS, as the different symptom domains of schizophrenia, especially negative symptoms, were reportedly have association with ToM (46–48). According to the remission criteria of schizophrenia (33) we measured three negative symptom items of PANSS: blunted affect, passive/apathetic social withdrawal and lack of spontaneity and flow of conversation. Results on negative symptoms and ToM suggest (46, 47) these symptoms may interfere with ToM tasks, especially with verbally mediated tasks having substantial verbal memory and expression demands. However, our patients exhibited only mild or no negative symptoms, which suggests that negative symptoms might not have influenced the performance in STT. The lack of the full PANSS however is still a limitation of the study, and future investigations should address this dimension. We did not use independent auditory verbal comprehension test to rule out the confounding effect of verbal comprehension. Although SST includes comprehension questions, the fact that persons with schizophrenia performed less accurate in terms of the comprehension questions compared to the control subjects highlights this limitation. Due to time restrictions we did not have the opportunity to test concurrent validity with other ToM measures in schizophrenia. In spite of the fact that the main purpose of this study was to test the applicability of the new SST with persons with schizophrenia, the lack of use of another ToM test as a comparison is yet another important limitation that should be addressed in future studies. While general intelligence was not assessed, the educational levels were compared in the two samples in order to limit the potential effects of IQ. The question of medication of the patient group also poses another limitation. All patients were on maintenance antipsychotic treatment medications and—while data on the effects of antipsychotics on ToM are limited—according to our current knowledge antipsychotics do not significantly influence patients' mental state reasoning abilities (18, 49).

### Conclusion

As a conclusion we found that our results lined up with the original Dodell-Feder et al. (1) findings and we found that reading fiction could be used as an assessment tool for explicit ToM skills in persons with schizophrenia. The patients performed more poorly in SST compared to healthy controls. The SST lacks the ceiling effect, and it is sensitive to explore the individual differences in ToM performance, and so it can be useful in planning psychosocial interventions. An important limitation of SST is the low sensitivity to measure spontaneous ToM and the relatively long administration time.

## Data Availability Statement

The datasets generated for this study are available on request to the corresponding author.

## Ethics Statement

The studies involving human participants were reviewed and approved by Committee on Medical Ethics, University of Pécs. The patients/participants provided their written informed consent to participate in this study.

## Author Contributions

JF: study design, data collection, interview scoring, data analysis, manuscript writing. ZP: statistical analysis, manuscript writing. EV: interview scoring, data analysis. TC: data analysis, manuscript writing OZ: pilot study, data analysis. TT: study design, psychopathology assessment, manuscript revision. RH: study design, psychopathology assessment, manuscript writing and revision.

## Funding

This study was supported by the National Research Program Grant No. NAP-A-II-II/12 (2017–2021) and the Hungarian National Excellence Centrum Grant 2018–2019.

## Conflict of Interest

The authors declare that the research was conducted in the absence of any commercial or financial relationships that could be construed as a potential conflict of interest.
